# Energy Drink Consumption in Europe: A Review of the Risks, Adverse Health Effects, and Policy Options to Respond

**DOI:** 10.3389/fpubh.2014.00134

**Published:** 2014-10-14

**Authors:** João Joaquim Breda, Stephen Hugh Whiting, Ricardo Encarnação, Stina Norberg, Rebecca Jones, Marge Reinap, Jo Jewell

**Affiliations:** ^1^Nutrition, Physical Activity and Obesity Programme, Division of Noncommunicable Diseases and Life-Course, World Health Organization Regional Office for Europe, Copenhagen, Denmark; ^2^WHO Country Office for Estonia, Tallinn, Estonia

**Keywords:** energy drinks, Europe, consumption, review, risks, health effects, policy

## Abstract

With the worldwide consumption of energy drinks increasing in recent years, concerns have been raised both in the scientific community and among the general public about the health effects of these products. Recent studies provide data on consumption patterns in Europe; however, more research is needed to determine the potential for adverse health effects related to the increasing consumption of energy drinks, particularly among young people. A review of the literature was conducted to identify published articles that examined the health risks, consequences, and policies related to energy drink consumption. The health risks associated with energy drink consumption are primarily related to their caffeine content, but more research is needed that evaluates the long-term effects of consuming common energy drink ingredients. The evidence indicating adverse health effects due to the consumption of energy drinks with alcohol is growing. The risks of heavy consumption of energy drinks among young people have largely gone unaddressed and are poised to become a significant public health problem in the future.

## Introduction

In 2006, almost 500 new brands of energy drinks were released worldwide (1). The energy drink industry is booming, with sales of energy drinks estimated to be over 12.5 billion USD in 2012, an increase of 60% from 2008 to 2012 (2). Energy drinks are relatively new to the wider soft drinks market, with the first energy drink launched in Japan in 1960. Energy drinks first appeared in Europe in 1987 before quickly expanding throughout the rest of Europe and appearing in the US in 1997 (2). While no standard definition of an “energy drink” is used in the scientific literature, it is commonly understood to be a non-alcoholic drink that contains caffeine (usually its main ingredient), taurine, vitamins, and sometimes a combination of other ingredients (such as guarana and ginseng, among others), marketed for its perceived or actual benefits as a stimulant, for improving performance and for increasing energy (2).

Although energy drinks are a relatively new class of beverage, they are quickly becoming as a central part of the partying subculture, particularly among young people who commonly mix energy drinks with alcohol (3–7). The full impact of the rise in popularity of energy drinks has not yet been quantified, but the aggressive marketing of energy drinks targeted at young people combined with limited and varied regulation have created an environment where energy drinks could pose a significant threat to public health (1, 4).

In 2011, the European Food Safety Authority (EFSA) commissioned a study to gather consumption data for energy drinks in 16 countries of the European Union. They found that 68% of adolescents (aged 10–18 years old), 30% of adults, and 18% of children (<10 years old) consumed energy drinks. Among adolescents, consumption varied from 48% in Greece to 82% in the Czech Republic. Among children, consumption varied from 6% in Hungary to 40% in the Czech Republic. The average consumption was 2 l in adolescents and 0.49 l in children (2).

With increasing consumption and an increase in the number of reported cases of adverse health effects associated with energy drink consumption, concerns have been raised both in the scientific community and among the general public about the health impact of these products. Despite this, there have been limited rigorous studies carried out in Europe on the risks associated with the increase in energy drink consumption, particularly among young people. The adverse health effects related to energy drink consumption and over-consumption are still highly debated from a scientific point of view (2), and this paper sets out to review the available literature on the associated health risks and policies related to energy drinks.

## Method

We searched the Cochrane Library, Plos One, and PubMed for relevant publications. We searched databases by using the terms *energy drinks* and *adverse effects* in the Medical Subject Heading (MeSH) database. The MeSH terms are part of a distinct vocabulary created by the National Library of Medicine to index articles for MEDLINE and PubMed that provides a consistent way to retrieve information using different terminology. We reviewed publications retrieved from this search and selected those that we judged to be relevant. We reviewed articles through June 2014 and did not exclude articles based upon date of publication due to lack of literature in this area. We also searched PubMed using a combination of the following terms: *risk, consumption, adverse health effects, policies, mixed drinks, alcohol*, and *Europe*. We included English language articles only. An attempt was made to limit the scope of our review to policy literature solely focused on the European region. However, due to a lack of literature, other regions were included in the review. We also excluded all publications focused on animal models.

## Risks Associated with Energy Drink Consumption

The health risks associated with energy drink consumption are primarily related to their caffeine content. A caffeine overdose can cause palpitations, hypertension, dieresis, central nervous system stimulation, nausea, vomiting, marked hypocalcemia, metabolic acidosis, convulsions (8), and, in rare cases, even death (9, 10). In adults, there is also an increased risk of arterial hypertension (11) and Type 2 diabetes (12), as high consumption of caffeine reduces insulin sensitivity (13). High-caffeine consumption among pregnant women increases the risk of late miscarriages, small for gestational age infants, and stillbirths (14).

Although some types of coffee can have caffeine levels comparable to energy drinks, coffee is typically consumed hot and consequently more slowly (4). Further, the proliferation of new brands of energy drinks has included some brands, which contain extreme caffeine levels much higher than mainstream brands as they try to establish themselves in the market (1). In Europe, the EFSA study showed that the estimated contribution of energy drinks to total caffeine exposure was 43% in children, 13% in adolescents, and 8% in adults (2). There are proven negative consequences of caffeine consumption among children and adolescents, including effects on the neurological and cardiovascular systems, which can cause physical dependence and addiction (15).

Consumption of energy drinks among adolescents is associated with other potentially negative health and behavioral outcomes such as sensation seeking, use of tobacco and other harmful substances, and binge drinking and is associated with a greater risk for depression and injuries that require medical treatment (16, 17). Recent literature has also found an increasing number of problems with behavior modification and cognitive capabilities in adolescents who use energy drinks (18).

Energy drink consumption may be a risk factor for alcohol dependence even if not mixed with alcohol (19).This phenomenon is hypothesized to be due to the neuropharmacologic effects of caffeine increasing the tendency for addiction (4). There is also an increased risk of obesity, due to the high-sugar content of energy drinks (20). A study in the US showed that dental cavities can result from the acidic pH and high-sugar content of products such as energy drinks (21), and another study showed that consumption of energy drinks can cause erosion and smear layer removal in the teeth, leading to cervical dentin hypersensitivity (22).

While caffeine is considered the main ingredient in energy drinks, there are often a number of other substances present. The most common of these include guarana, taurine, glucuronolactone, and B vitamins (23). As the acute and long-term effects of the combined consumption of many of these substances with caffeine are not well known, further studies are required to examine the potential for adverse health effects from energy drink consumption, particularly from long term, habitual consumption (24).

## Risks Associated with Consumption of Energy Drinks and Alcohol

The practice of mixing energy drinks with alcohol is on the rise (3, 25), with 71% of young adults (18–29 years old) who consume energy drinks, mixing them with alcohol (2).

There is an increasing amount of research linking energy drink consumption with high-risk behavior, particularly when combined with alcohol. A study of US college students found that those who reported combining energy drinks with alcohol were more likely to experience adverse consequences due to their own drinking compared to those who only drank alcohol. Adverse consequences included: being taken advantage of or taking advantage of someone sexually; riding with an intoxicated driver; and being hurt or injured (3). In Australia, energy drink consumers were more likely to have a higher breath alcohol concentration reading, to pre-drink and use illicit drugs, and to have engaged in risky behavior in the previous 3 months including involvement in a fight or drink-driving (26). Further studies from the US found a positive association between energy drink consumption and high-risk behaviors including marijuana use, fighting, sexual risk taking, failure to use seatbelts, taking risks on a dare, smoking, drinking, problems stemming from alcohol abuse, and illicit drug use (6, 27, 28). Another study from the US military indicated that soldiers who consumed energy drinks had a higher prevalence of suicidality and soldiers who combined energy drinks with alcohol had an even higher prevalence (29).

The consumption of high amounts of caffeine contained within energy drinks reduces drowsiness without diminishing the effects of alcohol resulting in a state of “wide awake drunkenness,” keeping the individual awake longer with the opportunity to continue drinking (4, 30). Studies have found that while the consumption of energy drinks with alcohol significantly reduces the subjective perceptions of some symptoms of alcohol intoxication including impairment of motor coordination, there is no actual reduction in the effects of the alcohol on the impairment of motor coordination, reaction time, or the breath alcohol concentration (31). A positive attitude and perception about alcohol mixed energy drinks also indicates higher consumption (32). Combining energy drinks and alcohol has also been associated with increased heavy drinking sessions and episodes of weekly drunkenness (3). A small randomized controlled trial in the US showed that energy drinks combined with alcohol seems to increase the motivation to consume greater amounts of alcohol compared to the same amount of alcohol alone (33). Research has continually shown the harmful risks associated with mixing energy drinks and alcohol; however, risks are still present when consuming energy drinks by themselves.

## Adverse Events Associated with Energy Drink Consumption

Adverse events resulting from energy drink consumption are generally caused by the sympathomimetic effects from an excess intake of caffeine^1^ (1, 34). As energy drinks have not always had their own unique tracking code in poison centers, there is a lack of information available for studies of energy drink over-consumption and associated adverse events (35). However, there have been a number of case reports indicating the potential for adverse health effects due to energy drink over-consumption.

In 2007, a man in Australia was reported to have suffered cardiac arrest after consuming seven to eight cans of an energy drink while taking part in vigorous physical activity (36). A Swedish study in 2006 identified a number of cases with severe symptoms and a number of deaths possibly linked to energy drinks (37). Iyadurai and Chung (38) reported on four cases in the US, where patients presented at emergency rooms after suffering new, adult-onset seizures and the only common finding was that all the patients had consumed large amounts of energy drinks. Once the patients abstained from consuming energy drinks, no further seizures were reported. Avci et al. (39) reported another case from the United States where a 28-year-old man consumed three 250 ml energy drink cans, 5 h before a basketball match. After playing for 30 min, he lost consciousness, suffered from cardiac arrest, and died 3 days later. While a causal relationship between the consumption of large amounts of energy drinks and new-onset seizures has not been confirmed, further research in this area would be prudent.

A retrospective review of calls made to a poison information center in Australia over a 7-year period found that 297 calls related to caffeinated energy drink exposure were recorded, with call numbers increasing from 12 in 2004 to 65 in 2010. The researchers raised the possibility that this was a significant underestimate due to the lack of adequate coding of energy drinks by the poison center studied and the fact that they were only able to access approximately 50% of the total calls to poison information centers in Australia (34). The National Poison Data System in United States (NPDS) recorded 4854 calls (0.2% of total calls) related to energy drinks over the year 2010–2011. Among the calls that led to more severe adverse effects, 39.3% involved alcohol mixed energy drinks. In all, 68.2% of the alcohol-related cases were individuals under the age of 20 (40). These studies demonstrate that energy drink consumption and toxicity is an extensive and growing problem in Australia and the United States and that a similar investigation into the European poison centers may be necessary.

## Marketing of Energy Drinks

Marketing of energy drinks focuses on their stimulant effects and perceived benefits such as increased performance, attention, stamina, and weight loss, which remain unproven (1). Energy drink advertising targets young males with a focus on promoting the psychoactive, performance-enhancing, and stimulant effects of energy drinks. The marketing of some brands even attempt to glorify drug use (1) with one brand going as far as advertising itself as “the legal alternative” to cocaine (41). Further, a study found that self-reported measures of masculinity and risk taking behaviors were positively associated with frequency of energy drink consumption (28). In 2010, the Food and Drug Administration of the US announced that caffeine was unsafe for use as an additive to alcoholic beverages and the Federal Trade Commission notified manufacturers that they were potentially engaged in the illegal marketing of unsafe alcoholic drinks (4).

Marketing campaigns that focus on improved performance, as well as a target market of children and adolescents and inadequate labeling, can increase the risk of caffeine intoxication from energy drink consumption (1). The aggressive marketing of energy drinks and the association of some brands with athletes and sporting events has led to many athletes consuming energy drinks before competitions to improve performance (42) or to recover expended energy after competition (5). In Europe, a study found that 41% of adolescents consumed energy drinks for physical activity purposes (2). Excessive caffeine consumption combined with strenuous physical activity can be dangerous (36), and the association of energy drinks with sports performance should be reconsidered.

## Existing Policies

Several countries have enacted measures to regulate the labeling, distribution, and sale of energy drinks that contain significant amounts of caffeine. Since 2004, European regulations have enforced additional caffeine labeling for energy drinks that contain at least 150 mg/l of caffeine (43). From 2014, these will be strengthened to ensure that all beverages with high-caffeine content or with caffeine added for its physiological effects will be labeled with the statement “High caffeine content. Not recommended for children or pregnant or breast-feeding women,” followed by the caffeine content expressed in mg/100 ml (2).

Concerns about the risks of excessive caffeine consumption previously led to outright bans on energy drinks in Denmark, Turkey, Norway, Uruguay, Iceland, and France (5), although the French government reluctantly removed its ban in 2008 following an assessment by EFSA, which found no definitive safety risk, taurine-related or not (44). Energy drinks can currently be sold in all EU Member States, although some national legislators have decided to take a more specific regulatory approach, including by setting rules for sales to minors. In Sweden, for example, sales of some products are restricted to pharmacies and sales to children (<15 years) are banned. Canada enforces warning labels that specify maximum daily consumption and include warnings about mixing energy drinks with alcohol (45). In Australia and New Zealand, energy drink manufacturers have previously bypassed regulations by classifying products as a “dietary supplement” to avoid caffeine limits of 80 mg/250 ml can (44). Finally, a “public health tax” was adopted in Hungary in 2012 that applies to caffeinated energy drinks, in addition to a range of other products and nutrients. The tax is levied on drinks containing >1 mg of methylxanthines or >100 mg of taurine per 100 ml at a rate of approximately €0.81/l (46). Energy drinks in developed countries remain largely unregulated (5), partially because of the long term and widespread consumption of beverages such as coffee and tea in which caffeine is a natural constituent (1).

## Recommended Policies

This review of the published literature on energy drinks identifies a number of policies that might be considered by policy makers as they move to minimize the potential for harmful effects from energy drink consumption.

There should be an evidence-based, upper limit for the amount of caffeine allowed in a single serving of any drink (4). While the majority of energy drinks that control the market do not contain excessive amounts of caffeine, there are an increasing number of energy drinks entering the market that have caffeine concentrations well above those of mainstream energy drinks (47). Setting a maximum limit for caffeine per serving of any energy drink throughout Europe could remove the extreme, highly caffeinated energy drinks from stores and protect the public’s health (47).

The restriction of sales to children and adolescents should be considered due to the potentially harmful adverse and developmental effects of caffeine on children (15). Health practitioners also need to be aware of the potentially dangerous consequences of excess caffeine consumption. Policies should ensure that health-care providers are equipped to educate families and children at risk on the potential consequences of excessive energy drink consumption and recognize the features of caffeine intoxication, withdrawal, and dependence (1). Diet and substance-use histories in primary health care should include screening for dangerous energy drink consumption, both alone and with alcohol (3, 12).

Energy drink manufacturers aggressively market their products to children, adolescents, and young adults. The absence of regulatory oversight in many countries has contributed to the aggressive marketing of energy drinks targeted primarily toward young males (1). Regulatory agencies should enforce industry-wide standards for responsible marketing of energy drinks and ensure that the risks associated with energy drink consumption are well known.

## Future Research

There is an on-going need for further research on the possible adverse effects of energy drink consumption in Europe. A harmonized approach is vital for data matching, which could lead to new findings about population groups that may be particularly at risk for adverse outcomes due to energy drink consumption. Further research is required to determine whether there is a causal link between energy drink consumption and adult-onset seizures (38).

A number of studies have shown that energy drink consumption is very high among adolescents and increasing among children (2, 12). More research is required to characterize the effects of long-term energy drink consumption, particularly among children and young adults, as well as the suitability of restriction options before widespread bans are put in place (44). Other areas of investigation related to children and adolescents include the contribution of energy drinks to the childhood obesity epidemic, psychiatric illness including attention deficit/hyperactivity disorder, as well as insomnia (48).

More research is needed that focuses on the practice of mixing alcohol with energy drinks in Europe, particularly among young people. The potential risk for injury or excessive intoxication in young people who consume energy drinks with alcohol is significant. More data are needed to determine the risk of alcohol poisoning as a result of consuming energy drinks with alcohol (4), as well as to identify the populations who are most at risk. The identification of policies that are effective in reducing the incidence of adverse events at the national level could ensure the successful implementation of similar policies in neighboring countries.

## Conclusion

From a review of the literature, it would appear that concerns in the scientific community and among the public regarding the potential adverse health effects of the increased consumption of energy drinks are broadly valid. The potential for acute caffeine toxicity due to consumption of energy drinks may be greater than other dietary sources of caffeine due to the variable and sometimes very high-caffeine content of energy drinks, in combination with the aggressive marketing to young and inexperienced consumers (1).

The potential health risks related to heavy consumption of these products have largely gone unaddressed. Furthermore, new developments in marketing are also aimed at increasing the perceived health functionality of energy drinks in order to gain acceptance in an increasingly health-driven society (47, 49, 50). As energy drink sales are rarely regulated by age, like alcohol and tobacco, and there is a proven negative effect of caffeine on children, there is the potential for a significant public health problem in future. To date, policy development has been limited. Where policies exist, they are yet to be systematically evaluated in terms of their impact on heavy energy drink consumption, particularly among children and young adults. From a cautionary viewpoint, further research and policy action is necessary to minimize the risk of harm from heavy and long-term energy drink consumption.

## Conflict of Interest Statement

The authors declare that the research was conducted in the absence of any commercial or financial relationships that could be construed as a potential conflict of interest.

## Footnotes

^1^Even as little as 50 mg of caffeine can induce tachycardia and agitation. In overdose, caffeine toxicity can mimic amphetamine poisoning and lead to seizures, psychosis, cardiac arrhythmias, and, potentially but rarely, death.
